# Editorial: *Helicobacter pylori* infection and antibiotic resistance: clinical, translational and experimental studies

**DOI:** 10.3389/fcimb.2023.1296784

**Published:** 2023-10-02

**Authors:** Abbas Yadegar, Ali Nabavi-Rad, Sinéad Marian Smith

**Affiliations:** ^1^ Foodborne and Waterborne Diseases Research Center, Research Institute for Gastroenterology and Liver Diseases, Shahid Beheshti University of Medical Sciences, Tehran, Iran; ^2^ Department of Clinical Medicine, School of Medicine, Trinity College Dublin, Dublin, Ireland

**Keywords:** *Helicobacter pylori*, antibiotic resistance, gastric microbiota, gastric ulcer, probiotic

*Helicobacter pylori* (*H. pylori*) is a Gram-negative recalcitrant oncomicrobe that infects over half of the world’s population. Infection with *H. pylori* is the primary cause of gastric and duodenal ulcers and gastric malignancies (Malfertheiner et al., 2023). Current guidelines suggest the test-and-treat strategy, which is the antibiotic-based eradication of *H. pylori* upon diagnosis (Malfertheiner et al., 2022). However, the development of antibiotic resistance mechanisms and the subsequent emergence of single-drug and multi-drug resistant *H. pylori* strains have made the achievement of successful eradication extremely challenging (Tshibangu-Kabamba and Yamaoka, 2021). Therefore, monitoring antibiotic resistance, elucidating resistance mechanisms and developing innovative treatment approaches have become of significant interest in this field of study. In this regard, the Research Topic of “*Helicobacter pylori* infection and antibiotic resistance: clinical, translational and experimental studies” appeared highly topical, presenting novel insights on the critical challenge of *H. pylori* antibiotic resistance and prospective treatment strategies.

Given the growing body of research around *H. pylori* infection, Yu et al. analyzed *H. pylori* research hot topics over the past decade by performing a bibliometric and visual analysis. They meticulously elaborated on the increasing trend in *H. pylori*-related gastric ulcer research, with China being the most prolific country in terms of publications in this area and the United States as the most influential country. A cross-sectional study by Jiang et al. demonstrated an updated situation of *H. pylori* antibiotic resistance in China. Although analysis of 2109 *H. pylori* clinical isolates presented an overall reduction of primary resistance rate over the past years, *H. pylori* strains are as yet highly resistant to metronidazole (67.2%), clarithromycin (36.0%), and levofloxacin (24.2%). Considering the recommendation that clarithromycin susceptibility testing should be performed prior to prescribing any clarithromycin containing *H. pylori* first-line therapy (Malfertheiner et al., 2022), developing simple and more rapid techniques for detecting *H. pylori* clarithromycin resistance seems essential (Alavifard et al., 2023).

*H. pylori* infection is associated with significant alterations of the gastric microbial and metabolic profile (Fakharian et al., 2022). Successful eradication of *H. pylori* could considerably restore the indigenous structure of the gastric microbiota; however, refractory *H. pylori* infection and persistent administration of various antibiotics further disturb the host microbial profile. Liu et al. demonstrated that compared to the *H. pylori*-positive control group, refractory *H. pylori* infection contributes to the enrichment of infectious disease-associated metabolic pathways in the gastric microbiota, such as energy metabolism, lipopolysaccharide biosynthesis, glutathione metabolism, and sulphur metabolism. Refractory *H. pylori* infection mainly featured in the colonization resistance of beneficial *Lactobacillus* bacteria in the stomach. Given the supplementation of various beneficial bacterial strains (probiotics), mainly *Lactobacillus* bacteria, as adjunctive therapies for *H. pylori* eradication (Nabavi-Rad et al., 2022; Nabavi-Rad et al., 2023), Kiattiweerasak et al. investigated the efficacy of *L. rhamnosus* and *L. helveticus* administration for *H. pylori* eradication in a double-blind, randomized, placebo-controlled study. Despite the similar eradication rates between the probiotic and placebo groups, probiotic treatment substantially reduced drug-related side effects including bloating, diarrhea, nausea, and bitter taste. Furthermore, the probiotic group presented an elevation in general health-related quality of life.

In conclusion, the published studies in this Research Topic discussed the significant importance of *H. pylori* antibiotic resistance and its remarkable impact on the host gastric microbial structure. These studies further elaborated the potential for microbiome-based therapeutics in improving *H. pylori* treatment and health-related quality of life. Nevertheless, antibiotic resistance mechanisms of *H. pylori* are yet to be fully elucidated and require further in-depth investigation.

## Author contributions

AY: Supervision, Writing – review & editing. AN-R: Writing – original draft. SS: Writing – review & editing.
